# *Wolbachia* Infection Decreased the Resistance of *Drosophila* to Lead

**DOI:** 10.1371/journal.pone.0032643

**Published:** 2012-03-05

**Authors:** Ling Wang, Chun Zhou, Zhen He, Zheng-Guang Wang, Jia-Lin Wang, Yu-Feng Wang

**Affiliations:** Hubei Key Laboratory of Genetic Regulation and Integrative Biology, College of Life Science, Central China Normal University, Wuhan, People's Republic of China; Tokyo Medical and Dental University, Japan

## Abstract

**Background:**

The heavy metal lead has been shown to be associated with a genotoxic risk. *Drosophila melanogaster* is a model organism commonly utilized in genetic toxicology testing. The endosymbionts — *Wolbachia* are now very common in both wild populations and laboratory stocks of *Drosophila*. *Wolbachia* may induce resistance to pathogenic viruses, filarial nematodes and *Plasmodium* in fruit fly and mosquito hosts. However the effect of *Wolbachia* infection on the resistance of their hosts to heavy metal is unknown.

**Methodology/Principal Findings:**

Manipulating the lead content in the diet of *Drosophila melanogaster*, we found that lead consumption had no different effects on developmental time between *Wolbachia*-infected (Dmel *w*Mel) and –uninfected (Dmel T) flies. While in Pb-contaminated medium, significantly reduced amount of pupae and adults of Dmel *w*Mel were emerged, and Dmel *w*Mel adults had significantly shorter longevity than that of Dmel T flies. Lead infusion in diet resulted in significantly decreased superoxide dismutase (SOD) activity in Dmel T flies (P<0.05), but not in Dmel *w*Mel flies. Correspondingly, lead cultures induced a 10.8 fold increase in malonaldehyde (MDA) contents in Dmel T larvae (P<0.05). While in Dmel *w*Mel larvae, it resulted in only a 1.3 fold increase. By quantitative RT-PCR, we showed that lead infused medium caused significantly increased expression level of *relish* and *CecA2* genes in Dmel T flies (P<0.01). Lead cultures did not change dramatically the expression of these genes in Dmel *w*Mel flies.

**Conclusions/Significance:**

These results suggest that *Wolbachia* infection decreased the resistance of *Drosophila* to lead likely by limiting the production of peroxides resulted from lead, thus being unable to activate the immunological pathway in the host to prevent them from lead damage. This represents a novel *Wolbachia*–host interaction and provides information that researchers working on *Drosophila* toxicology should take in consideration the presence of *Wolbachia* in the stocks they are analyzing.

## Introduction

*Wolbachia* are obligatory Gram-negative bacteria infecting a great number of species of arthropods and nematodes. It has been estimated that up to 66% of insect species are infected with *Wolbachia* [1]. The widespread success of *Wolbachia* is thought to be largely attributed to their ability to manipulate their hosts' reproduction which may selectively favor infected females. This manipulation can be accomplished through a variety of strategies such as sperm-egg cytoplasmic incompatibility (CI), parthenogenesis, feminization of males, and male-killing [2]. Furthermore, *Wolbachia* infection may also affect the olfactory response, life span, and immunity of their hosts [3]–[5]. For example, *Wolbachia* strain *w*MelPop reduces the longevity of its *Drosophila melanogaster* host [4] and also has been shown to halve life span when artificially transferred to mosquito *Aedes aegypti* [6]. Recent studies revealed that *Wolbachia* may function in protection against pathogenic viruses, filarial nematodes and *Plasmodium* in fruit fly and mosquito hosts [5], [7]–[11]. Infection of adult *Drosophila* with *Drosophila C virus* (DCV) can induce 100% mortality within 5∼6 days. In contrast, the flies infected with both *Wolbachia* and DCV died within 13∼14 days [7]. Furthermore, the presence of *Wolbachia* in *Aedes aegypti* inhibits the development of filarial nematodes, resulting in significantly reduction in the numbers of third larval stage worms in the mosquito [5]. In addition, highly significant reductions in *Plasmodium* infection intensity were observed in the *w*MelPop-infected *Anopheles gambiae*, indicating that *Wolbachia* infection inhibits the development of *Plasmodium* in the mosquito [10]. However, the effect of *Wolbachia* infection on the resistance of their hosts to heavy metal is unknown.

Lead (Pb) is one of the most abundant heavy metal pollutants in the environment. It is considered to be one of the most hazardous chemicals for humans and animals, since it may induce a broad range of acute or chronic behavioral, biochemical and physiological abnormalities. Pb-induced lipid peroxidation of cellular membranes has been demonstrated to play a critical role in the oxidative damage of liver [12]. Pb may produce serious immunotoxicity to phagocytic activity as well as cellular and humoral immunity, resulting in increased host susceptibility to infection, or tumorigenesis [13]–[15]. One of the major mechanisms concerning the toxicity of Pb is attributed to its ability to generate reactive oxygen species (ROS), which results in oxidative stress [16], [17]. Studies *in vitro* have shown that NF-κB, AP-1, MEK, and JNK may be important regulators of Pb-induced signaling in gene expression mediating inflammatory response and immunomodulation [18]. In addition, the treatment of macrophages with Pb results in disregulation of the production of pro-inflammatory cytokines, such as tumor necrosis factor alpha (TNF-α), interleukin 1alpha (IL-1α) and interleukin 6 (IL-6) [19]. Probably it is by this signal pathway that the excessive production of ROS associated with Pb exposure affects the viability of both lymphocytes and macrophages, thus damage the immune functions of organisms.

*D. melanogaster* is commonly employed in genetic toxicology testing. Its metabolic activity that may activate pro-mutagens and pro-carcinogens is analogous to that of the liver in mammals [20]. Therefore, it has been used as a model organism to study the mechanisms of mutagenesis [21], [22]. However *Wolbachia* infections in *D. melanogaster* are extremely common now in both wild populations and long-term laboratory stocks [23]. In order to investigate the influence of *Wolbachia* infection on the resistance of *D. melanogaster* to heavy metal, we compared the survival, growth and longevity between *Wolbachia*-infected and -uninfected flies reared in lead-supplemented medium. We observed that *Wolbachia* infection reduced the survival rate of *Drosophila* living in Pb-contaminated diet. We then analyzed the possible mechanisms by which *Wolbachia* affect the resistance of the host to Pb and found that *Wolbachia* infection limited the oxidative stress and restrained activation of immune related genes induced by lead culture in *Drosophila*. This exhibits a new *Wolbachia*–host interaction and provides a reminder that researchers studying on *Drosophila* toxicology should take in consideration the presence of *Wolbachia* in the stocks they are experimenting.

## Results

### *Wolbachia* infection limited the survival of *Drosophila* reared in lead-contaminated medium

**Figure 1:**
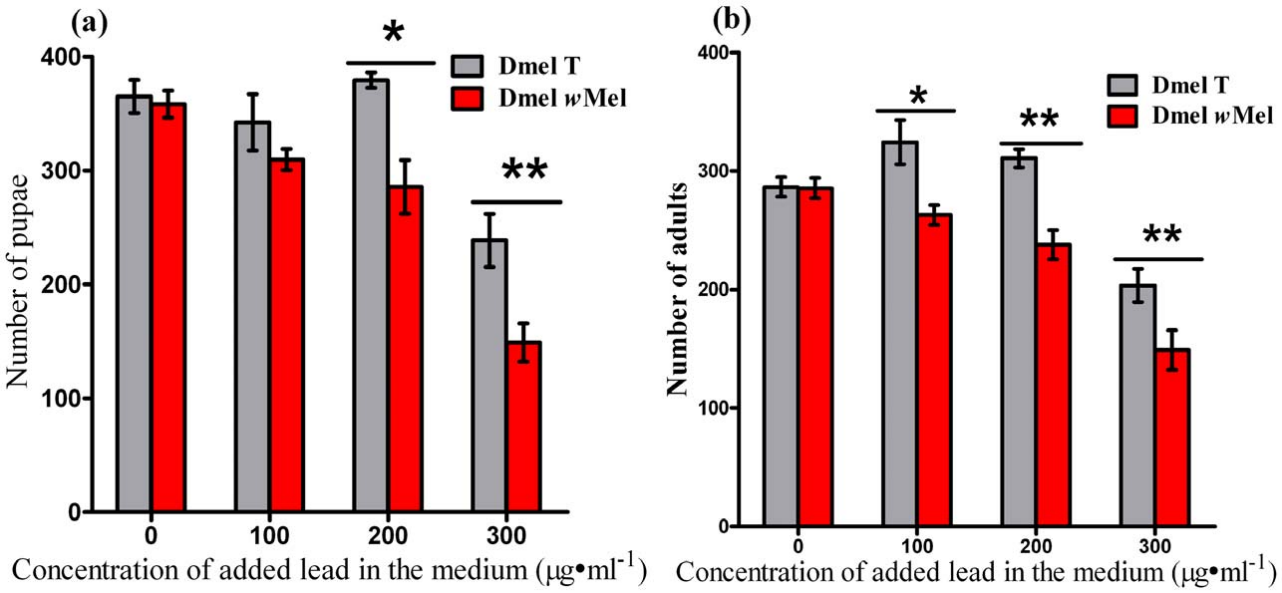
Influence of *Wolbachia* infection on survival of *Drosophila* reared in Pb-contaminated medium. (**a**) Number of pupae of Dmel *w*Mel and Dmel T emerged from each group. (**b**) Number of adults of Dmel *w*Mel and Dmel T emerged from each group. Bars = standard error; “*” indicated P<0.05; “**” indicated P<0.01.

In order to compare the effect of lead on the development of *Wolbachia* infected and uninfected *Drosophila*, we first arranged the same number of 3∼4-day-old flies to lay eggs for 9 hours in the medium supplemented with 0 µg⋅ml^−1^ (as control), 100 µg⋅ml^−1^, 200 µg⋅ml^−1^ and 300 µg⋅ml^−1^ of lead acetate, respectively. Then we counted the numbers of pupae and adults developed in each group. Comparison of the numbers of pupae and adults showed no significant differences between Dmel *w*Mel and Dmel T living in regular medium (infused 0 µg·ml^−1^ of lead). However with the increasing of lead loaded in the medium, the amount of pupae and adults emerged were significantly different between Dmel *w*Mel and Dmel T flies. Comparing with Dmel T, the amount of pupae emerged decreased significantly in Dmel *w*Mel (P<0.05 for 200 µg·ml^−1^ group, P<0.01 for 300 µg·ml^−1^) (Figure 1a). Similarly, the numbers of eclosed Dmel *w*Mel adults were also significantly less than that of Dmel T (P<0.05 for 100 µg⋅ml^−1^ group, P<0.01 for 200 and 300 µg·ml^−1^ groups) (Figure 1b).

### *Wolbachia* infection had no effect on the developmental time of *Drosophila* reared in Pb-contaminated food

**Figure 2:**
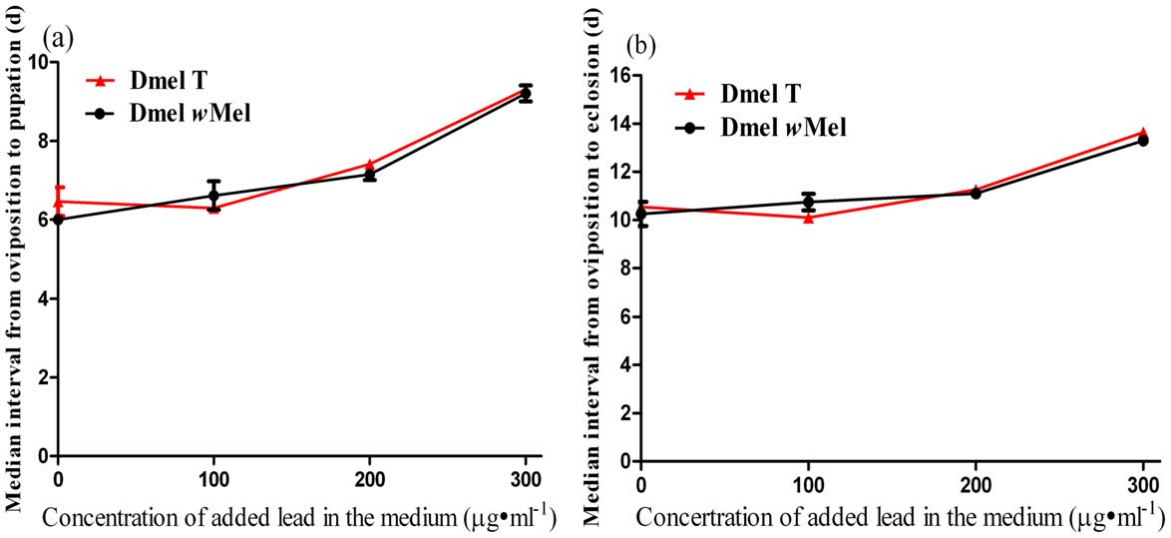
Effect of *Wolbachia* infection on the developmental time of *Drosophila* reared in Pb-contaminated food. (**a**) Median interval from oviposition to pupation. (**b**) Median interval from oviposition to eclosion. Bars = standard error.

The lead cultures had an extended developmental time from oviposition to pupation and to eclosion for both Dmel *w*Mel and Dmel T flies. However, for the food groups infused with the same concentration of lead, there was no significant differences between Dmel *w*Mel and Dmel T flies in developmental time (P>0.05) from oviposition either to pupation or to eclosion (Figure 2a, b). This indicated that the Pb-contaminated food resulted in delayed fly development, but *Wolbachia* infection had no effect on the developmental time of *Drosophila*.

### *Wolbachia* infection decreased the longevity of *Drosophila* after lead consumption

**Figure 3:**
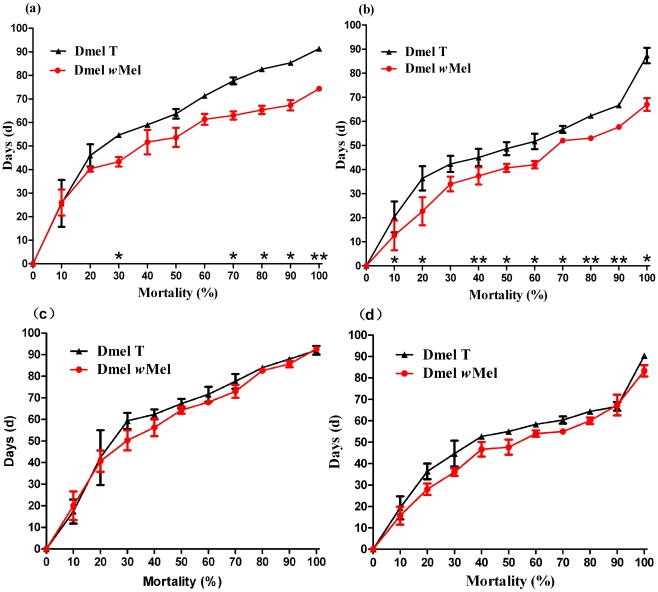
The longevities of *Wolbachia*-infected and -uninfected flies living in the medium infused either 300 µg·ml^−1^ (a, b) or 0 µg·ml^−1^ (c, d) of lead. (**a, c**) Female adults. (**b, d**) Male adults. Bars = standard error; “*” indicated P<0.05; “**” indicated P<0.01.

Low concentration of lead in diet did not cause significant difference of longevity between Dmel *w*Mel and Dmel T flies (data not shown). However, high concentration of lead added in food (300 µg·ml^−1^) resulted in significantly shorter life span in Dmel *w*Mel flies than that in Dmel T flies (Figure 3). The longest life span of Dmel *w*Mel females was 74.33±0.33 days, significantly shorter than that of Dmel T females (91.33±0.67 days) (P<0.01) (Figure 3a). The average life span of Dmel *w*Mel females was 54.63±2.43 days, also notably shorter than that of Dmel T females (65.73±1.47 days) (P<0.05). For males, the maximum longevity of Dmel *w*Mel flies was 67.00±2.65 days (the average was 41.90±2.16), also dramatically shorter than that of Dmel T flies (87.33±3.18 days, with average of 51.73±2.83) (P<0.05) (Figure 3b). The infection of *w*Mel *Wolbachia* did not influence the longevity of the flies, since in regular medium (containing 0 µg·ml^−1^ of lead) there were no significant differences of life span between Dmel *w*Mel and Dmel T flies for both females (Figure 3c) and males (Figure 3d).

### The effect of *Wolbachia* infection on SOD activities and MDA contents in *Drosophila* reared in lead overloaded medium

**Figure 4:**
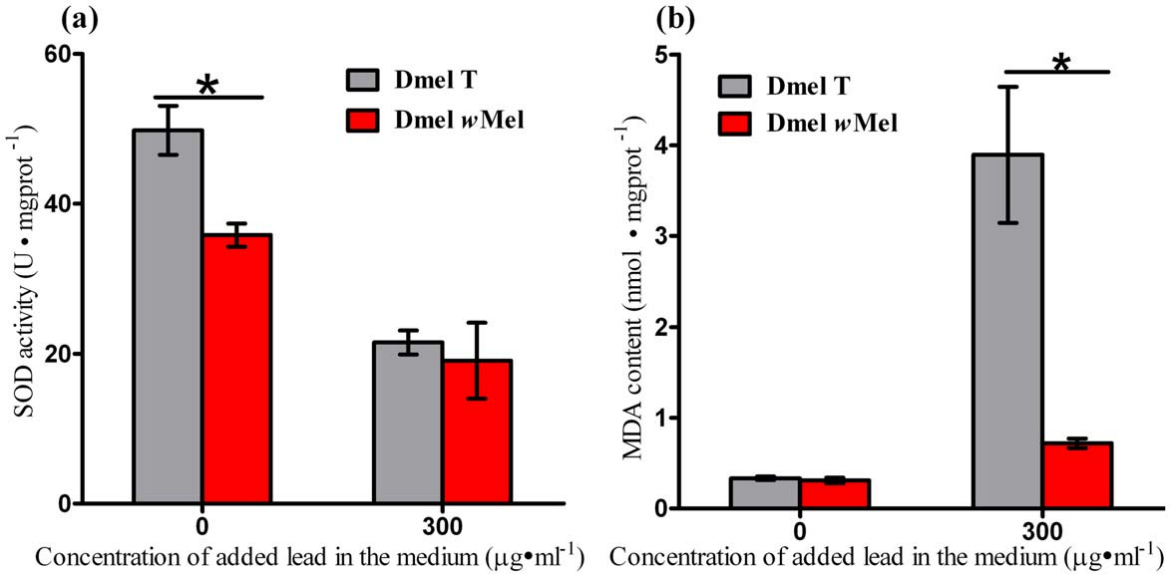
*Wolbachia* infection limited peroxidation in *Drosophila* larvae cultured in the medium infused 300 µg·ml^−1^ of lead. (**a**) Comparison of SOD activities in Dmel *w*Mel and Dmel T larvae after challenge with lead. (**b**) Comparison of MDA contents in Dmel *w*Mel and Dmel T larvae after challenge with lead. Bars = standard error; “*” indicated P<0.05.

Since the toxicity of lead mainly lies in its inducing production of ROS [16], [17], to investigate whether the variation of viability of *Drosophila* after lead challenge is involved in oxidative stress, we measured the superoxide dismutase (SOD) activities and malonaldehyde (MDA) contents in the 3^rd^ instar larvae. *Wolbachia* infection significantly decreased the SOD activity in *Drosophila* larvae when reared in the regular medium (P<0.05) (Figure 4a). High concentration of lead (300 µg·ml^−1^) infused medium caused markedly reduction of SOD activity in Dmel T larvae (P<0.01). However, for Dmel *w*Mel larvae, the same concentration of lead supplemented in the medium did not result in significant difference of SOD activity (P>0.05). There was no significant difference of SOD activities between *Wolbachia*-infected and -uninfected fly larvae when cultured in the medium infused 300 µg·ml^−1^ of lead (P>0.05) (Figure 4a).

MDA contents had no significant difference between the Dmel *w*Mel and Dmel T larvae when cultured in regular medium. Lead supplementation in diet resulted in a 10.8 fold increase in MDA contents in Dmel T larvae (P<0.05). While in Dmel *w*Mel larvae, lead infusion in medium induced only a 1.3 fold increase. In the medium loaded 300 µg·ml^−1^ of lead, MDA content in Dmel *w*Mel larvae was notably lower than that in Dmel T larvae (P<0.05) (Figure 4b).

### The effect of *Wolbachia* on the immune-related pathway of *Drosophila* after challenge with lead

**Figure 5:**
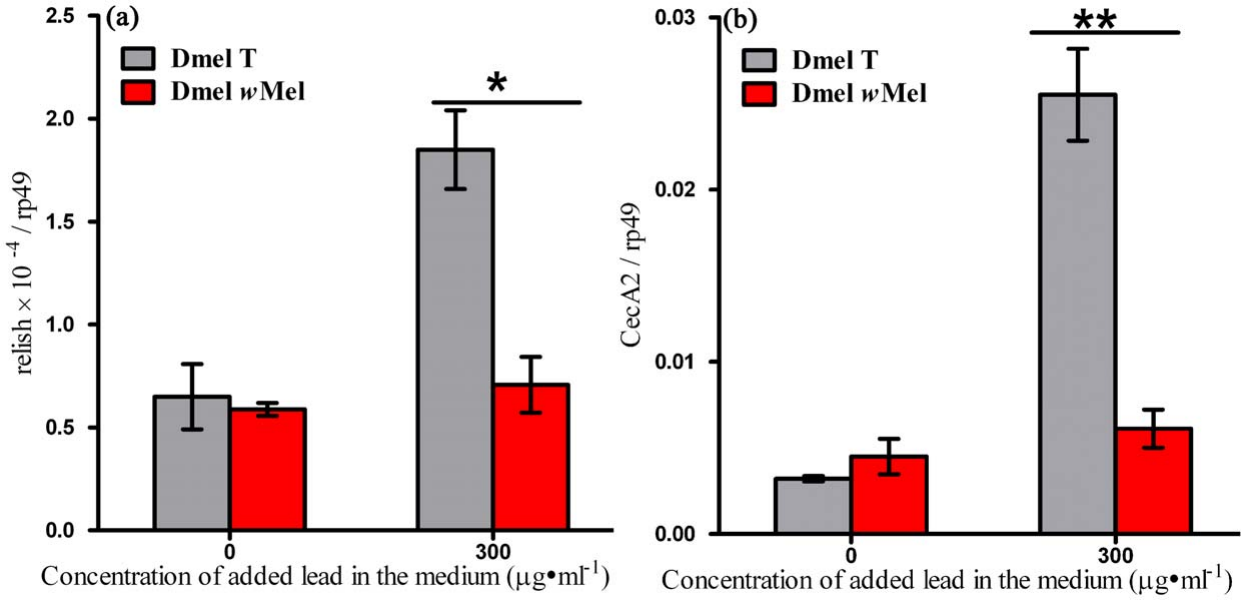
Effect of *Wolbachia* on the expressions of *relish* (a) and *CecA2* (b) genes in *Drosophila* larvae cultured in Pb-contaminated medium. Bars = standard error; “*” indicated P<0.05; “**” indicated P<0.01.

It was reported that both *Wolbachia* infection and lead exposure had effects on the immune system of the animals [5], [24]. To investigate the influence of *Wolbachia* on *Drosophila* hosts in Pb-contaminated environment, we assayed the expression level of *relish* gene which is associated with immune-related IMD pathway in insects. We found that there was no significant difference between Dmel *w*Mel and Dmel T larvae under regular culture conditions. However, when 300 µg·ml^−1^ of lead was added in the medium, the expression level of *relish* gene was significantly increased in Dmel T larvae (P<0.01), whereas it remained consistent in Dmel *w*Mel larvae, thus the expression level of *relish* in Dmel *w*Mel larvae was notably lower than that in Dmel T larvae (P<0.05) (Figure 5a). To further demonstrate the effect of *relish* expression level on lead resistance, we examined the viability of *relish^E20^* (*relish* null mutant) raised in the medium containing 300 µg·ml^−1^ of lead. We observed that the eclosion rate (emerged adults/eggs) of relish mutants was 4.0%, apparently lower than that of control (W^1118^), which was 37.15%. In an attempt to further understand the effect of *Wolbachia* on the immune-associated pathway of *Drosophila* following challenge with lead, we then studied the transcription of antimicrobial peptide marker gene *Cecropin A2* (*CecA2*). As is evident in Figure 5b, lead contamination induced significantly increased expression level of *CecA2* in Dmel T larvae, but not in Dmel *w*Mel larvae.

## Discussion

Several studies demonstrated that *Wolbachia* infection increased the resistance of *Drosophila* and mosquito hosts to pathogenic viruses, filarial nematodes and *Plasmodium* [5], [7], [10], [11], [25]. For example, presence of *Wolbachia* reduced the load of viruses and delayed virus-induced mortality in *D. melanogaster*. While removal of *Wolbachia* with tetracycline renders flies more sensitive to RNA viruses [7], [8]. Moreover, *Wolbachia* may not only inhibit viral replication, dissemination and transmission, but also restrain the development of filarial nematodes and *Plasmodium* in the mosquito hosts [5], [9]–[11]. Conversely, recent work on *Wolbachia*-mediated antibacterial protection revealed that *Wolbachia*-infected *Drosophila* was not protected from pathogenic Gram-negative bacteria [26]. In this study we investigate whether *Wolbachia*-infected flies are resistant to heavy metal. We show that in fruit flies, *Wolbachia* infection impairs seriously the survival for both *Drosophila* larvae and adults under lead-contaminated conditions, since significantly reduced amount of pupae and adults are emerged in Dmel *w*Mel flies compared with Dmel T flies when the medium was supplemented high concentration of lead (Figure 1). Moreover Dmel *w*Mel adults have significantly shorter longevity compared with Dmel T flies when reared in lead – infused medium (Figure 3). This suggests that *w*Mel *Wolbachia* decreased the resistance of *Drosophila* to the heavy metal pollutant — lead.

One major mechanism associated with the toxicity of lead is owing to its ability to produce ROS [16], [17]. Current studies have shown that infusing 300 µg·ml^−1^of lead in the medium results in markedly reduction of the activity of SOD (the primary enzyme for radical scavenging, a process responsible for defense against oxidative stress) and increase of MDA (marker of lipid peroxidation) contents in Dmel T larvae, indicating that lead cultures results in oxidative stress in Dmel T larvae. This is consistent with previous reports in humans and rats exposed to lead [27], [28]. The inhibition of various enzymes resulted from lead exposure might impair antioxidant defenses and causes the cells to be more susceptible to oxidative damage [29]. However for Dmel *w*Mel larvae, growing in the medium added the same concentration of lead limits the alteration of the SOD activity and MDA contents. This suggests that lead cultures limit oxidative stress in Dmel *w*Mel larvae. *Wolbachia* have been known to be highly prevalent symbionts and infect over 66% of insect species [1]. Since the toxic oxidants produced by immune cells are primarily directed to kill microorganisms, hence they likely have developed several strategies to avoid host defense in order to allow them to persist within the host cells as microbial pathogens often do [30]. Oxidative stress may activate NF-κB [31], thus activate the immune system [32], therefore *Wolbachia* might rely on some special systems to limit oxidative stress in *Drosophila* host reared in Pb-contaminated medium so as to keep them living in the host cells. This is in agreement with the results *in vitro* when iron was overloaded in the medium [33]. In *Wolbachia*-infected and uninfected *A. aegypti* cells, ferritin expression was not significantly different under standard culture conditions. However, when iron was added in the medium, the ferritin level remained constant in *Wolbachia*-infected cells, whereas it increased significantly in *Wolbachia*-uninfected mosquito cells [33]. Since an excess of iron in the cells is harmful by catalyzing ROS, and ferritin can contribute to iron homeostasis and reduction of oxidative stress [34], hence the constant level of ferritin expression in *Wolbachia*-infected cells after iron overloaded in the medium indicates that *Wolbachia* may interfere with iron also in a way that limits oxidative stress.

In insects, the inducible expression of antimicrobial peptides is controlled by the Toll and IMD signal transduction pathways [35], [36]. A number of peptidoglycan recognition proteins (PGRPs) act as receptors for Gram-negative bacterial peptidoglycan. The adaptor protein IMD interacts with the receptor and converge signals to downstream components including the transcription factor Relish, which is homologous to NF-κB1 (p105) in mammals [35]–[37]. Since *Wolbachia* are Gram-negative bacteria, we focus on IMD pathway to explore the effects of *Wolbachia* infection on the resistance of *Drosophila* hosts to lead. Our results show that in the regular medium the expression level of *relish* between Dmel T and Dmel *w*Mel larvae did not exhibit significant difference. Although several evidences both *in vitro* and i*n vivo* have shown that *Wolbachia* infection up-regulated the expression of immune related genes [5], [10], [11], [38] and probably thus increased the resistance of the hosts to pathogenic viruses and filarial nematodes, yet both mosquito hosts and the mosquito cells that are used in the experiments are artificially transinfected with *Wolbachia*. However, in *D. simulans* and *A. albopictus*, naturally occurring *Wolbachia* was found neither constitutively to induce nor to suppress the transcription of various inducible antibacterial genes [39]. In this study, *D. melanogaster* that we used are naturally infected with *w*Mel *Wolbachia* (Dmel *w*Mel) for long time, which could provide an explanation that there is no significant difference of *relish* gene expression level between *Wolbachia* infected and uninfected flies reared in the regular medium. In current study we also show that lead supplement causes significantly increased expression of two key genes in immune-related pathway: *relish* and *CecA2* in Dmel T larvae. *Relish* is a NF-κB related protein gene and is associated with immune-related IMD pathway in insects. *Cecropin A2* (*CecA2*) is antimicrobial peptide marker gene in downstream of immune-related pathway in insects. Hence this result suggests that lead cultures might induce the activation of immune system in Dmel T larvae. However, in Dmel *w*Mel larvae lead did not dramatically alter the expression levels of *relish* and *CecA2* genes. This is probably due to the limitation of oxidative stress induced by lead in Dmel *w*Mel larvae. Since oxidative stress may activate NF-κB, and then induce the activation of immune-related pathway to resist damages [32], therefore we speculate that in *Wolbachia*-uninfected flies, lead cultures results in oxidative stress, which activate the immune-related genes, thus activate the immune pathway, which is probably helpful to resist damage caused by lead. However, *Wolbachia* infection limits the oxidative stress induced by lead consumption, thus cannot activate the immune system to help counteracting the lead damage. How the activation of immune system helps the flies to resist lead damage needs to be further investigated.

## Materials and Methods

### Fly stock

All flies were maintained on standard cornmeal diet at a temperature of 25(±1) °C with a 10 h∶14 h (light∶dark) cycle and were reared under non-crowded condition [40]. *Wolbachia*-infected Dmel *w*Mel (*D. melanogaster* Brisbane nuclear background with introgressed *w*Mel from YW) was kindly provided by Prof. Scott O'Neill at Monash University, Australia. Cured Dmel *w*Mel (designated Dmel T) were subsequently generated by tetracycline treatment following established protocols [41] and confirmed to be *Wolbachia*-free by PCR using the primers from *Wolbachia* surface protein (*wsp*) gene (data not shown). Relish mutant (w^1118^; Relish^E20^ e^s^) was a gift from Prof. Yan Li at the institute of biophysics, CAS.

### Observation of development

Adults were fed on standard cornmeal by the ratio of females versus males of 3∶1 for 3 days. Then flies were transferred to conical flasks (150 ml) with food supplemented with 0 µg·ml^−1^ (control), 100 µg·ml^−1^, 200 µg·ml^−1^ or 300 µg·ml^−1^ lead acetate, respectively. For each repeat, 18 female and 6 male adults were used. Adult flies were allowed to lay eggs for 9 hours in the medium. Then the developmental time of the flies in each group was calculated every 12 hours (0.5 d) from the date that the egg was laid to the date when half pupae were emerged or to the date when half adults were eclosed. The numbers of emerged pupae and adults were tallied daily.

For *relish^E20^* mutants, 200 eggs were collected and put in 150 ml conical flask containing 50 ml of medium infused with 300 µg·ml^−1^ lead acetate for development. Considering that this mutant is produced based on W^1118^ flies, we used W^1118^ flies as control. The eclosed adults were recorded and used to calculate the eclosion rate (emerged adults/eggs).

Two-day-old female and male adult flies were transferred to vials with the medium containing 0 µg·ml^−1^, 100 µg·ml^−1^, 200 µg·ml^−1^ or 300 µg·ml^−1^ of lead acetate. Flies were transferred to the corresponding fresh food every 4 days. Every repeat contains 30 flies. Mortality was recorded daily until all flies died.

### Measurement of SOD activity and MDA content

The SOD activity and MDA content were determined by using the SOD and MDA detection kits purchased from Nanjing Jiancheng Bioengineering Institute (Nanjing, China) according to the manufacturer's instructions which had been described by Yang et al [42]. SOD activity was expressed as Units (U)· mg protein^−1^. MDA content was expressed as nmol of MDA produced per mg protein.

### Quantitative reverse transcriptase PCR (qRT-PCR)

Total RNA was extracted from the 3^rd^ instar larvae of *w*Mel and *w*Mel T cultured in medium with 0 µg·ml^−1^ or 300 µg·ml^−1^ of lead acetate using Trizol (Invitrogen). DNA contamination was removed with RNase-free DNase I (Takara). The first-strand cDNA was synthesized from around 2 µg of total RNA using M-MLV reverse transcriptase (RT) (Invitrogen) and oligo dT15 primer (Takara) at 37°C for 50 min. Specific primers were designed based on flybase and the sequences are as follows: relish-F: 5′-CAGGTGCGGCTCTGCTTTG-3′, relish-R: 5′-GGTTTGCTCAGGCGGACG-3′; CecA2-F: 5′-TAAAACCACCATGAACTTCT-3′, CecA2-R: 5′-CCAACACGTTCGATTTTCTT-3′; rp49 (using as reference gene)-F: 5′-CTAAGCTGTCGCACAAATGG-3′, rp49-R: 5′-TAAACGCGGTTCTGCATGAG-3′. Quantitative PCR was performed using a Miniopticon system (BioRad) with a Platinum SYBR Green qPCR superMix (Takara). The reaction volume was 20 µl, containing 10 µl SYBR Premix Ex Taq (2×), 0.3 µl of forward and reverse primer (10 mM), respectively, 7.4 µl ddH2O and 2 µl of cDNA template diluted by 10-fold. The qPCR procedure was consisted of 95°C for 2 min, followed by 95°C for 10 s, 58°C (for *relish* and *rp49*) or 57°C (for *CecA2*) for 15 s and 72°C for 10 s per cycle for 40 cycles, then a melting curve analysis was carried out by a slow increase (0.2°C/s) from 55°C to 98°C, in purpose of examining if there were primer-dimers or nonspecific amplification. The relative expression ratio of gene for samples was calibrated against *rp49* gene using the 2^−ΔCT^ calculation method: ΔC_T_ = C_T, gene_−C_T, rp49_.

### Statistics analysis

Results are presented as means ± SE (n = 3). Differences among means were analyzed by Student t-test. Differences were regarded as statistically significant when *P<0.05.
